# Intersection of Structural Conditions that Contribute to Healthcare Access After Release from Prison: Qualitative Findings from the SCHARP Study

**DOI:** 10.1007/s11606-025-10060-4

**Published:** 2025-12-10

**Authors:** Megan A. Morris, Ingrid A. Binswanger, Kathy Gleason, Carly Ritger, Morgan Ford, Irene V. Blair, Rebecca Hanratty, Stacie L. Daugherty

**Affiliations:** 1 Department of Medicine, University of Colorado School of Medicine, Aurora, CO, USA; 2 Adult and Children Center for Outcomes Research and Delivery Sciences (ACCORDS), University of Colorado School of Medicine, Aurora, CO, USA; 3 Kaiser Permanente Colorado, Institute for Health Research, Denver, CO, USA; 4 Kaiser Permanente Bernard J. Tyson School of Medicine, Health Systems Science, Pasadena, CA, USA; 5 University of Colorado Boulder, Department of Psychology and Neuroscience, Boulder, CO, USA; 6 Department of Medicine, Denver Health, Denver, CO, USA; 7 New York, USA

**Keywords:** structural conditions, people released from prison, primary care

## Abstract

**BACKGROUND:**

People re-entering the community after prison face poor health outcomes and challenges accessing care. Structural conditions in healthcare systems may contribute to these problems.

**OBJECTIVE:**

Understanding how multi-level conditions intersect and potentially compound one another to impact care delivered to patients recently released from prison.

**DESIGN:**

Qualitative content analysis.

**PARTICIPANTS:**

Participants were 17 healthcare leaders, 27 clinicians, 25 frontline staff, and 20 community members (*n* = 89). Sixteen community members had lived experience with incarceration; clinicians and frontline staff were in behavioral health, primary care, and emergency medicine.

**MAIN MEASURES, OR APPROACH:**

We used interview guides that incorporated a conceptual model linking organizational structural conditions to health outcomes. We coded interview transcripts with deductive and inductive codes and analyzed them using qualitative content analysis.

**KEY RESULTS:**

People recently released from prison experience limits to accessing primary care. Findings revealed interrelated themes at the patient, healthcare, and prison system levels. Patients released from prison have heightened social needs (e.g., lack of financial resources or transportation), which significantly impact their ability to seek care and experiences with care. Patients’ lack of skills to navigate the healthcare system and healthcare staff’s lack of specialized training and experience, and healthcare system unavailability of patient information and medical history, can all contribute to gaps or difficulties for people getting care. Potential solutions identified include increasing access to primary care, support for insurance enrollment, availability of a primary care appointment upon release, access to medical clinics specialized in transition from incarceration or peer navigators, and better partnership between the Department of Corrections and healthcare systems.

**CONCLUSIONS:**

To improve the health of persons recently released from prison, solutions need to address structural conditions in both healthcare and society. Solutions also require collaboration between and within healthcare systems, policy makers, and community organizations.

## Graphical Abstract

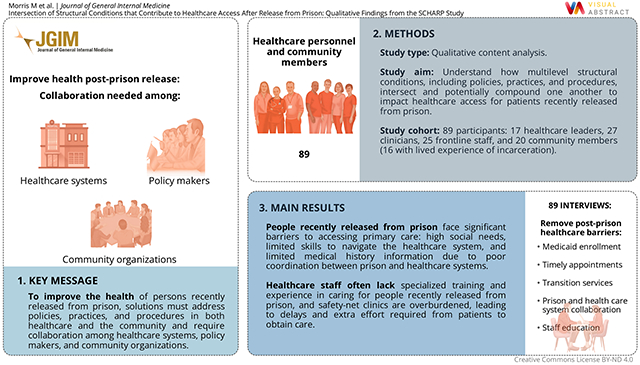

## INTRODUCTION

People released from prison face high mortality and morbidity upon community reentry.^1–5^ To date, most research exploring health outcomes and care access post incarceration focuses on behavioral health and substance use, with fewer studies focusing on physical health conditions. The studies focusing on physical health report that people released from prison have high rates of chronic conditions, including hypertension and diabetes, and less access to outpatient primary care.^6^

People recently released from prison face challenges accessing healthcare in the community, including mistrust and discrimination in healthcare settings,^7,8^ social needs (e.g., housing, employment), multiple medical and psychiatric comorbidities,^8–11^ and navigating the complex healthcare and insurance system.^8,12,13^ Furthermore, structural conditions, or the policies, practices, and attitudes at the healthcare system, healthcare team, and prison system levels, likely play a role in healthcare access for people released from prison. Understanding how multi-level conditions intersect and potentially compound one another is an important step in improving healthcare access for this population.

We conducted a qualitative study that involved interviews with healthcare system leaders, clinicians, frontline staff, representatives of community-based organizations, and people with lived experience. We sought to understand how policies, practices, and attitudes around access to physical healthcare contribute to and affect healthcare access for people recently released from prison.

## METHODS

### Study Design and Setting

This was an a priori qualitative aim of the Structural Conditions in Healthcare After Release from Prison (SCHARP) study. Guided by conventional qualitative content analysis methodology,^14,15^ we engaged participants across academic, community, and safety-net healthcare systems, community organizations, and people with lived experience in the Denver/Aurora metro area of Colorado. Conventional qualitative content analysis methodology was appropriate for our study question as the goal was to derive meaning directly from the interview transcript data. Around 40% of people from Colorado prisons are released to the Denver/Aurora Metro area. By focusing on a specific geographic area, we were able to conduct an in-depth and comprehensive exploration of the multi-level factors within the community. The Kaiser Permanente Interregional Institutional Review Board (KPiIRB #1867395) approved this study.

### Study Team

The study team included a PhD qualitative researcher (MAM), two physicians with health services research training (SLD and IAB), a PhD health equity researcher (IVB), and two master’s-trained qualitative analysts (KG and CR). Interviewers (MAM, KG, and CR) had no established relationships with the participants. Any study team member with a relationship with a participant (e.g., worked within the same organization) was blinded to participants’ identification and responses. Prior to beginning the study, the team participated in a pseudo-bracketing process in which they discussed their previous experience, assumptions, and biases regarding the topic and the study participants. Throughout, team members continued to discuss how these factors may influence the conduct of the study.

A community advisory board (CAB) of nine individuals who represented healthcare and community organizations and three CAB members with lived experience was developed to oversee and provide input throughout the study. We identified potential CAB members based on referrals from healthcare and outreach to community orgs focused on reentry. These connections helped us identify people with lived experience. Potential CAB members were interviewed to explain the role and ensure fit. Final members were selected by the CAB chair and the research team. We tried to include a broad representation of types of organizations, ensuring inclusion of people from frontline roles and minimizing hierarchy amongst members (e.g., no healthcare leadership such as Chief Medical Officers), and different sex and race representation. The CAB chair is independent of the team members conducting the research activities.

The CAB met either in-person or virtually every 4 months. The CAB provided input on the study design, including reviewing and giving feedback on the interview guides. They additionally assisted with identifying potential participants and suggesting recruitment methods. Finally, they provided input on the preliminary findings, including ensuring that the findings were consistent with their experiences.

### Study Participants

Using a snowball sampling strategy, we worked with health system, community organization, and CAB contacts to identify and recruit individuals within each target group. Participants in healthcare systems included leaders (executives involved in policy decisions, medical directors, and program officers), clinicians (physicians and advanced practice practitioners in Emergency Medicine, Behavioral Health, and Primary Care), and frontline staff (social workers, case managers, access specialists). We also interviewed representatives from community organizations and the Department of Corrections involved in post-release programs. Finally, we recruited people with lived experience. Recruitment continued until preliminary analyses indicated thematic saturation.

### Interviews

Interviews of 30 to 60 min were conducted via phone or video conferencing and were audio recorded. Participants provided verbal consent. The team developed the semi-structured interview guide based on existing literature and input from the CAB. The guide was pilot tested with a few initial participants and modified as needed.

### Analysis

Audio recordings were professionally transcribed verbatim. The team (MAM, KB, and CR) entered the transcripts into Atlas.ti (vs. version 23) and used an inductive process to develop the codebook. This process included an independent review of a subset of transcripts, meeting to reconcile transcripts, developing an initial codebook, and continuing iterations until a final codebook was agreed upon. The remaining transcripts were coded by a team member, with 20% of the transcripts double coded to ensure consistent code application. Coded transcripts were analyzed within and across participant groups to identify major and minor themes.

## RESULTS

A total of 89 people were interviewed between March and October, 2023. Of the participants, 69 represented healthcare systems, 12 represented community organizations, and 16 were people with lived experiences (see Table 1 for participant demographics). Participants reported that people recently released from prison experience challenges accessing physical healthcare, including but not limited to not scheduling appointments, missing appointments, using emergency or urgent care unnecessarily, experiencing gaps in care, and not filling medication prescriptions.

**Figure 1:**
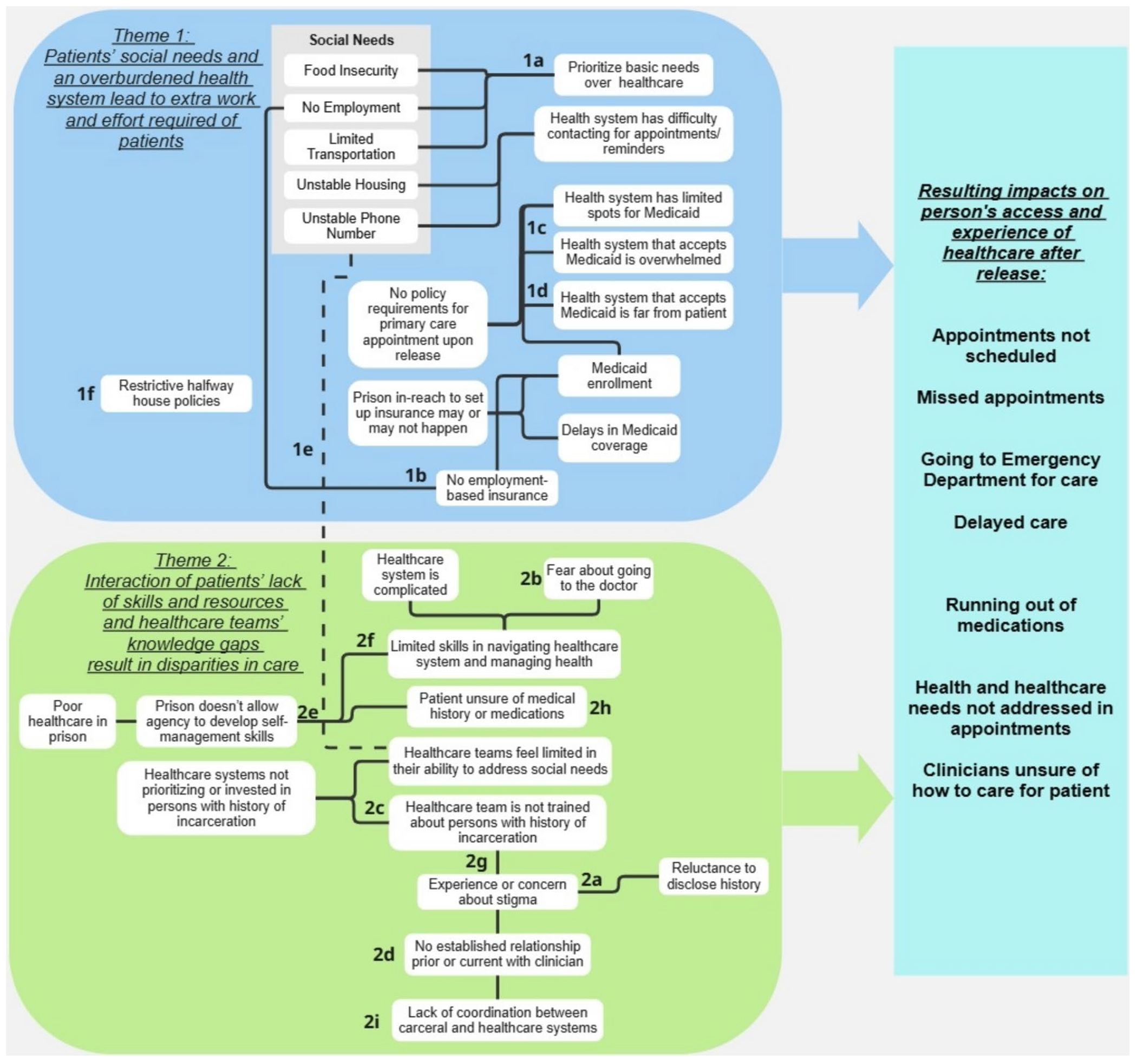
Map of intersecting conditions affecting healthcare experiences of persons after release from prison.

To understand contributors to these poor outcomes, participants described conditions at the state, healthcare system, clinical team, patient, and prison system levels that intersect and often compound each other. The relationship web of how these conditions intersect is complex and difficult to disentangle. Figure 1 is an example of how these conditions can relate and contribute to each other, based on the participants’ narratives. The following two themes describe examples of complex interactions as described by the participants. Throughout the themes, we will identify the conditions at the different levels. It is likely that there are many more connections between the different factors and there are likely factors not represented in these figures. Therefore, Fig. 1 should be considered a graphical representation of the themes. Throughout each theme, we reference the section of the figure discussed in the text.

### Theme 1: Patients’ Social Needs and an Overburdened Healthcare System Lead to Extra Work and Effort Required of Patients

People recently released from prison have high levels of social needs, which significantly impacts their ability and experiences with seeking care (patient-level). Participants discussed the work required of people once they are released to secure basic needs, including obtaining housing, transportation, employment, and food, as well as establishing themselves in the community (e.g., obtain a driver license, etc.). To survive, many prioritize securing basic needs over obtaining healthcare services or managing their health (Fig. 1, 1a). Additionally, many do not have the financial resources to pay for healthcare services not covered by insurance.

But I mean, my biggest barrier is transportation. […] I spent four to six hours on a bus every day. After pulling 10 hours at work or going where I’m going, it generally takes me almost an hour and a half, two hours to get anywhere out here. It’s not simple, especially with the way prices keep going up and rent, cost of living’s expensive, like to provide food and everything else for yourself, like it’s getting a little chaotic. (Person with lived experience)

Once a person is able to or requires care, the interaction of their social needs with healthcare system factors can complicate their ability to seek care. For example, participants reported that Medicaid is often the only insurance option for people recently released as they are unemployed and do not have access to employer-based insurance (Fig. 1, 1b). Participants reported that the majority of people released from prison in Colorado were enrolled in Medicaid, in large part due to state policy requirements (state-level). According to participants, while patients are often enrolled in Medicaid prior to release, variable release dates and other factors resulted in some needing enrollment support once released (prison-level).

Participants, particularly those who represented healthcare organizations, reported that non-safety-net healthcare systems often limited the number of patients, particularly new patients, they accepted with Medicaid or no insurance (healthcare system-level) (Fig. 1, 1c). This requires extra work for the patient to navigate the healthcare system to identify a clinic that accepts Medicaid and has availability. Consequently, people recently released often sought care within safety-net healthcare systems. Due to an overburdened safety-net healthcare system, there were often long delays in scheduling.

*If you call <Organization>’s appointment line and try to get new patient appointment, it’s generally somewhere between like a four- and ten-week wait, and that also requires persistence to follow up on it. It’s not like someone’s going to hold your hand four weeks down the road and make you do it*. (Clinician, primary care)

Further adding to the work required of the patient, participants reported that many people recently released had limited transportation and did not always live close to clinics associated with the safety-net healthcare system (patient and healthcare system levels) (Fig. 1, 1d).

*A lot of the <Organization > clinics are full or they don’t take Medicaid patients at this time or something and so they expect these patients to be farmed out to places like <Safety Net Hospital> or something like that, but maybe they live in <city> and they don’t actually have a lot of access to transportation or finances for meds or whatever. And instead of us having a mechanism to help them stay here and get care here because all their other social needs are sort of met in this immediate community, we expect them to compensate for our lack of resources, which is a huge, huge problem for patients*. (Frontline staff, emergency department)

Clinician participants reported the desire to assist people with addressing their social needs, but felt restricted within their role and with the resources available (healthcare team-level) (Fig. 1, 1e).

*We see so many people in and out of the ED [emergency department] throughout the day and trying to provide them resources and trying to engage them in wanting to make a change. […] At the end of the day, the resources are the resources, and I hate to say we’re just a hospital and just an emergency department, but we don’t have control over what’s out in the community*. (Frontline staff, emergency department)

Finally, transportation challenges and overburdened safety-net clinics were compounded by release requirement restrictions (prison-level) (Fig. 1, 1f). Multiple participants reported that people under correctional supervision in the community (i.e., under locked confinement part of the day or night) have barriers to seeking care. For example, a person on correctional supervision and living in a halfway house would get permission to attend an appointment during a specific time period. If a clinic was behind in the schedule, the person might be in violation of their time restrictions if they waited to be seen.

*And so when people come out, they’re still under a lot of supervision. They still have limited rights. They could get called at any moment and need to call and say where they are. If they to go get a lab or an X-ray, they have to call to get permission to do that, say, “I’m going to be this late.” There’s a lot of hassles. You have to get permission to be able to go to the doctor. There’s lockdown times. When you get to a halfway house for two to three weeks, some people can’t leave for any reason whatsoever. So you’re just going to lapse on your medications. It’s built into the policies*. (Clinician, primary care)

### Theme 2: Patients’ Lack of Skills and Resources and Healthcare Teams’ Knowledge Gaps Interact and Result in Disparities in Care

Participants reported that people released from prison often do not have the skills to navigate the healthcare system due to fear of discrimination, lack of experience with healthcare, and low self-efficacy (patient-level). Multiple participants stated that people released from prison experience significant stigma and discrimination, which can extend into the healthcare setting (Fig. 1, 2a). Consequently, patients may lack trust in the healthcare institution and healthcare teams. Participants with lived experience discussed a general fear of going to the doctor and asking for help (Fig. 1, 2b). These experiences and perceptions are exacerbated in the context of complex healthcare systems and healthcare teams that are unprepared and untrained to care for people recently released (healthcare system and healthcare team-level) (Fig. 1, 2c).

Many people recently released have little experience with the healthcare system prior to incarceration, with many not having health insurance or an established relationship with a primary care clinician (Fig. 1, 2d).

*And the truth is, is that with the incarcerated population, especially younger people, they didn’t have medical insurance and medical coverages before they went to prison because of the lifestyle. […] They didn’t have medical care, they didn’t have insurances, some of them were not working, they were involved with selling drugs and such. When they’re coming out of the system, it’s a whole new thing for them. It’s a whole new way to have to approach life and the healthcare and especially if they’ve been locked up long term*. (Community organization and person with lived experience)

Participants reported that the prison system does not allow for patient agency (prison-level) (Fig. 1, 2e). While in prison, people are not taught to take ownership of their own health and healthcare issues. Once released, people do not have the skills to care for themselves and their health.

*The system of incarceration really creates a learned helplessness for folks. And I don’t mean that in any way that is negative towards those people […] That is how that system is set up. You have no self-efficacy. You are told when to wake up, when to eat, you are housed […] And so it’s a system that really grinds people down, treats people like children at the best and in the worst doesn’t treat people like human beings. And so people internalize that and then when they get out, they don’t have the skills nor do they necessarily believe that they are worth receiving those skills*. (Frontline staff, primary care)

The lack of self-advocacy skills becomes acutely problematic when attempting to navigate the complex healthcare system (Fig. 1, 2f).

*The biggest problem though I think for guys getting out is navigating and getting to the place, finding a bus route, getting there and not getting impatient in the line, getting in the hospital and navigating where to go next. […] sometimes, you just get discouraged and you say ‘Well, I’ll put it off. I don’t need to do it right now. I’ll get it.’ And they keep putting it off until there’s an issue*. (Community organization, person with lived experience)

Participants reported that consequently, this population can become frustrated and use disrespectful language when interacting with healthcare teams, who can then perceive the person as hostile and uncooperative and are reluctant to assist the patient.

*But because of her incarceration, she was unable to communicate appropriately. So when she was calling and kept getting ‘no’, then she was frustrated and angry and cussing them out and hanging up on them and not able to even get an appointment scheduled due to frustration […] Other gaps is I’m not saying because you were incarcerated, you can talk to people like that. But people have to understand the frustration of hearing ‘No, no, no, no,’ when you know you need help*. (Community organization)

Participants who worked within healthcare organizations noted that their employer did not provide specific training or support for staff and clinicians regarding caring for people with a history of incarceration (healthcare system and team-level). Consequently, participants thought that staff and clinicians may not be sensitive or accommodating to patients with a history of incarceration (Fig. 1, 2g).

*There’s so much stigma around incarceration that people are scared of a former prisoner. They think that they deserve less care. […] They think that there’s something wrong with the person, that they’re dangerous. That happens quite a bit*. (Leadership, clinician)

Finally, participants reported that people recently released were often unaware or unsure of their medical history or current medications (Fig. 1, 2h). Clinician participants reported that there was a lack of coordination between the Department of Corrections and community healthcare systems, which resulted in a lack of sharing of patients’ records (Fig. 1, 2i). The combination of patients’ lack of knowledge of their medical history with a lack of sharing of medical records led to clinicians being uncertain on how to best care for their patients (patient, healthcare system, healthcare team, and prison-level).

*It’s real hard to get records. So, if you are going through recommendations and you recommend a colonoscopy, and it’s not uncommon for somebody to tell you that they had a colonoscopy while they were incarcerated. […] to really make a recommendation for when their next colonoscopy should be, we want to see the actual paperwork or path or something. And that can be difficult to obtain. And medications too. It’s usually a guessing game as to what medicines. They can often tell you what medicines but not doses*. (Clinician, primary care)

#### Potential Solutions.

To support people who have recently been released from prison and to promote equitable access to healthcare, participants identified five solutions and initiatives that currently have or have the potential to improve access. (See Table 2.) First, participants stressed the need to continue to work towards enrolling all people in Medicaid prior to release, as this assists in the timelier establishment of care. Second, participants recommended policies that require prompt primary care appointments once a patient is released. Third, participants discussed enrolling people recently released into transition clinics and/or having peer navigators available to alleviate the burden and work on patients. Fourth, multiple participants reported the need for increased partnerships and collaborations between the Department of Corrections and community healthcare systems. Lastly, participants reported the need for improved education for healthcare teams on the needs of people with a history of incarceration.

## DISCUSSION

Informed by 89 interviews, we identified intersecting conditions at the patient, healthcare team, healthcare system, and prison levels affecting access to primary care for people recently released from prison. For example, we identified that patients’ high burden of social needs and an overburdened health system negatively interacted, leading to extra effort required of patients and delayed access to healthcare. Similarly, patients’ lack of experience and skills navigating healthcare systems and healthcare teams’ knowledge gaps contribute to barriers in accessing healthcare. Solutions suggested by participants included enrollment in Medicaid, scheduling of primary care appointments, use of transition navigators, healthcare team education, and improved coordination between healthcare and prison systems. Given the high rates of chronic conditions and an aging prison population,^16^ healthcare access following release from prison is an important component in improving health outcomes for people recently released. Our findings are an important step in identifying potential solutions to improve healthcare access for this understudied population.

Some conditions identified by our study participants as negatively affecting access to care, including high social needs and discrimination in the healthcare system, are not unique to this study. Our study demonstrates that these barriers persist, despite being described for the past decade.^7–13,17,18^ An important contribution of this study is not simply naming barriers but exploring their relationships and including the perspective of healthcare organizations and teams, which may be critical in identifying solutions. An overarching study theme was the interconnectedness of the multiple levels of conditions. For example, participants identified how patients’ social needs, including lack of transportation, were compounded by being restricted to seeking care at safety-net clinics that were located far from the patient. This was further influenced by the fact that people released from prison are often on Medicaid because they are unemployed and do not have access to employer-based insurance, and that some healthcare systems limit the number of Medicaid patients they accept, requiring people to obtain care at a safety-net clinic. Understanding the relationships of how the conditions intersect and compound suggests that solutions to improving access to care will require multi-component interventions.

An important finding was that the solutions provided by the participants all addressed conditions at the healthcare system, healthcare team, and prison levels, not the patient level. This is likely due to a shift in recognizing the importance of structural conditions and how individual patients will always be limited in what they are able to do if the systems and policies are not designed to support them.^19^ Many of the identified patient-level conditions, including high social needs, are directly related to upstream systemic issues such as poverty. While not directly explored in our study, research demonstrates that these structural conditions have a downstream negative effect on health and healthcare outcomes.^20,21^

Participants described the work required of patients to access care. Because the healthcare system is not built to support unique population needs, including those released from prison, individual patients are left to compensate. For example, participants noted the long wait times for primary care appointments, which require a skillset and persistence to navigate the healthcare system to find a clinician who can see them in a timely manner. People released from prison are at a disadvantage as they likely did not enter into prison with those skills, nor are they given opportunities to build those skills in prison. The prison system is a structured setting with limited opportunity for agency or building self-advocacy skills. Addressing the structural conditions, along with equipping patients with skills, is likely necessary for sustainable change.

There are a few notable limitations of the study. First, we focused on one community, the greater Denver/Aurora, CO area, and the findings might not be generalizable to all communities. We focused on one community to attempt to comprehensively identify the multiple factors within a community that affect access to care. Additionally, even with our high number of interviews with diverse partners, it is possible that we did not capture all experiences and perspectives.

As identified in this study, the challenges accessing healthcare experienced by people released from prison are a result of a complex set of structural conditions that are often engrained in our healthcare system, healthcare teams, and prison system. Identifying long-term and sustainable solutions will require addressing these structural conditions as well as equipping people released from prison.

## Figures and Tables

**Table 1:** Participant Characteristics

	Total *N* = 89
Age	
18–39	25
40 and over	63
Missing	1
Sex	
Female	53
Male	35
Other	1
Race/ethnicity	
White	61
Black/African American	15
Hispanic	4
Other/multiple races	9
Prefer not to answer	2
Position/role	
Healthcare leadership	17*
Clinicians	27
Frontline staff	25
Community organization and Department of Corrections staff	12^†^
People with lived experience	16
Clinical area represented (for healthcare leadership, clinicians and frontline staff)	
Primary care	29
Emergency	13
Behavioral health	15
Administration/coordination/specialty	12

* Includes 9 clinicians,

† includes 8 persons with lived experience

**Table 2:** Solutions Identified by Participants

Solution	Quotes	Details
Enrollment in Medicaid upon release	*You’re supposed to, on the day of release, be able to activate your insurance straightaway. And that takes an incredible amount of organization. So the Department of Corrections in Colorado put real effort and a significant amount of resources to trying to increase the Medicaid enrollment and change the whole process of release. Release date is a variable moving target, very hard to say when someone’s going to be released. […] Went from 10 or 12% of people having Medicaid upon release when they first saw us to 80%. (Clinician, behavioral health)*	To be able to access primary and specialty care once released, people need to have health insurance. Due to lack of employment, Medicaid enrollment is often the only option
Policies requiring timely primary care appointments	…*make sure that at before somebody is released, that they have a follow up visit, ideally with a PCP or if they’ve got some chronic issue, if it needs specialty care. So to make sure there’s at least a follow up visit scheduled before somebody is released. (Leadership, clinician)*	Policies requiring timely appointments will require clinics to have appointment slots available for people recently released
Transition clinics with navigators	*People, they really need a sort of safe landing zone to help them through that transition [out of incarceration]. And I think our clinics could be a better partner to that than we are now […] It does seem to me, that more comprehensive wraparound program that’s there to help them transition, then medical care could be an appropriate partner to that. (Clinician, primary care)*	Navigating the healthcare system to identify a clinic that accepts Medicaid and that is accessible to the patient, requires extensive work and effort on the part of the patient. Transition clinics and patient navigators can help by providing support to the patient and alleviating the patient’s work
Improved collaboration between healthcare systems and Department of Corrections (DOC)	*I think the biggest bang for our buck would really be to create partnerships with the DOC and have peer support specialists and people with lived experience and social workers, be ready to route those patients to where they need to go upon release. (Leadership, behavioral health)*	Improved collaboration will assist with transition out of prison and integration into society. Collaboration could include sharing of medical records, which would allow clinicians to better understand patients’ medical history and provide better care
Improved education for healthcare teams	*I think just kind of awareness and education. And so providing education not only to kind of care management, behavioral health, but also providers and nursing and making sure everybody’s aware of different considerations for this population and some things that are helpful or might not be helpful. (Frontline staff, behavioral health)*	Equipping healthcare teams will improve their knowledge of the unique needs of people released from prison, including accommodations when a patient is struggling to navigate the healthcare system
